# Comparative Study of Gut Microbiome in Urban and Rural Eurasian Tree Sparrows

**DOI:** 10.3390/ani14233497

**Published:** 2024-12-04

**Authors:** Shuai Yan, Yu Zhang, Ji Huang, Yingbao Liu, Shaobin Li

**Affiliations:** 1 College of Life Sciences, Yangtze University, Jingzhou 434025, China; 2 Key Laboratory of Southwest China Wildlife Resources Conservation (Ministry of Education), China West Normal University, Nanchong 637009, China

**Keywords:** intestinal microbes, sparrows, urbanization, 16SrRNA, habitat, rural

## Abstract

Gut microbiota play a significant role in various physiological functions, including digestion, nutritional metabolism, and host immune function. The composition of these gut microbes is largely influenced by habitats. This study examines the gut microbiota of the Eurasian tree sparrow (*Passer montanus*) inhabiting rural and urban environments to understand the effects of habitat variation on microbial composition. We captured 36 rural and 29 urban adult tree sparrows and observed minor differences in body mass but substantial differences in foraging microhabitats between the two groups. Fecal samples from adult males with similar body mass were selected for a gut microbiome analysis to mitigate potential confounding effects, resulting in 20 successfully sequenced samples. The analysis disclosed disparities in gut microbiota diversity and composition between rural and urban sparrows. The urban group demonstrated slightly higher alpha diversity and distinct dominant phyla and genera compared to the rural group. Additionally, differences in the relative abundance of potentially pathogenic bacteria were observed between the groups. Several potentially pathogenic bacteria (e.g., TM7, *Staphylococcus*, *Helicobacter*, and *Shigella*) were more abundant in the urban group, suggesting that tree sparrows may act as transmission vectors and develop stronger immune systems. This could potentially facilitate pathogen dissemination while also contributing to the natural cycling of nutrients and maintaining ecosystem health in urban environments. The beta diversity analysis confirmed structural differences in microbial communities, implicating habitat variation as a contributing factor. Furthermore, the LEfSe analysis emphasized significant differences in gut bacteria abundance (across two phyla, three classes, six orders, seven families, and eight genera) between urban and rural sparrows, with predicted functional differences in metabolic pathways. Notably, lipid metabolism was enriched in urban sparrows, indicating enhanced lipid synthesis and metabolism in urban habitats. In conclusion, this study underscores the profound influence of habitat on the gut microbiota composition and functional potential in tree sparrows. Our findings highlight that urbanization alters the gut microbes and, consequently, the physiological functions of bird species.

## Simple Summary

We investigated the impact of habitat variation on the gut microbiota of the Eurasian tree sparrow (*Passer montanus*) by comparing individuals from rural and urban environments. We captured and analyzed fecal samples from both rural and urban adult male tree sparrows, focusing on those with similar body masses to control for potential confounding factors. The findings reveal differences in the diversity and composition of gut microbiota between the two groups. Urban sparrows showed slightly higher alpha diversity and had different dominant microbial phyla and genera compared to rural sparrows. Notably, urban sparrows had a higher abundance of certain potentially pathogenic bacteria, suggesting they might serve as vectors for disease transmission and have stronger immune systems. We also found differences in metabolic pathways, with urban sparrows showing enhanced lipid metabolism. Overall, this study highlights the impact of urbanization on the gut microbiota and physiological functions of tree sparrows.

## 1. Introduction

Gut microbiota are crucial to the physiological processes of the host, influencing the intestinal structure, digestion, immune function, and adaptations to environmental changes [1,2,3]. Understanding these microbial dynamics is essential for gaining insights into how these organisms contribute to health and adaptation [4,5], thereby illuminating broader ecological and evolutionary processes. It has been suggested that animals and microbes co-evolve, with habitat significantly influencing the intraspecific variation in gut microbial communities [6,7]. The gut microbiome of vertebrates is primarily shaped by their habitat and diet [8,9,10], with niche sharing among hosts potentially leading to microbiota convergence [11,12].

Birds, characterized by diverse life histories, feeding habits, and migration patterns, are particularly sensitive to environmental changes such as urbanization [13]. Urban environments, which are markedly different from rural areas due to increased human activity, altered landscapes, and modified ecological dynamics [14], often provide more abundant and diverse food resources for local avian species compared to rural habitats [15]. While extensive research has been conducted on avian life history and adaptation between rural and urban populations [16,17,18,19], studies focusing on the avian gut microbiota in these environments remain limited. Some previous studies have investigated differences in the gut microbiota of urban and rural birds by using simulated urban or rural environments/diets instead of examining actual urban and rural environments [20,21]. Other studies compared the gut microbiota of rural and urban individuals without controlling for sex or body mass [20,22,23]. The results from these studies have revealed that the effects of urbanization on gut microbiomes are not uniform—with some studies finding higher microbial diversity and others finding lower diversity in urban hosts, leaving uncertainty about how urban habitats shape gut microbial communities.

Urbanization has been shown to affect the gut microbiome composition of host species, though the patterns remain unclear [24,25]. In this study, we focus on the Eurasian tree sparrow (*Passer montanus*), a human-commensal species [26], to investigate the poorly understood differences in gut microbiota between rural and urban sites. This globally distributed, non-migratory species is closely associated with anthropized environments and inhabits both urban and rural areas [27]. Representing a typical avian group adapting to diverse ecological conditions near human settlements, they are suitable for investigating the impacts of urbanization on gut microbiota. The impact of urbanization on the gut microbiome of Eurasian tree sparrows remains largely unexplored. This study aims to compare the gut microbiota of urban and rural Eurasian tree sparrows, hypothesizing that urbanization alters gut microbial composition, with higher bacterial diversity in urban hosts compared to rural ones. The findings will provide insights into changes in the intestinal microbial flora of tree sparrows from rural to urban habitats and their broader adaptation to urban environments.

## 2. Materials and Methods

### 2.1. Bird Capture and Fecal Sample Collection

**Figure 1:**
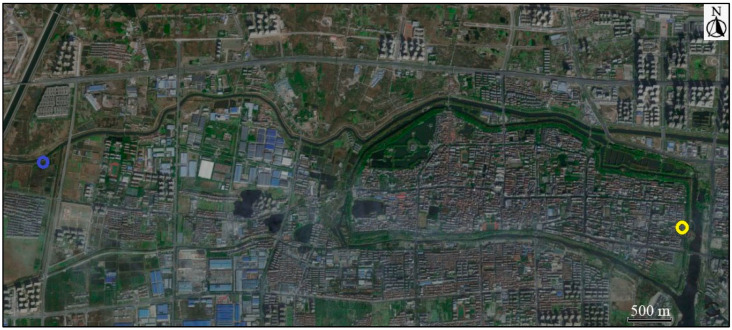
Sampling sites in satellite image from Baidu Map (left blue circle: rural site; right yellow circle: urban site).

Fieldwork was conducted in Jingzhou City, Hubei Province of China, from November 2021 to January 2022. Two sites with distinct habitats were selected for sample collection: an urban site near the city center characterized by an expansive built-up area covering over 60% of the landscape (30°21′12.56″ N, 112°08′37.78″ E) and a rural site near farmland (30°20′38.30″ N, 112°12′50.50″ E; Figure 1) where built-up areas are notably sparse, occupying less than 40% of the terrain.

The Eurasian tree sparrow is a widely distributed and dominant bird species in our study areas [19]; this species is sedentary in both sites [19], and their foraging distance is typically within 2 km [27] (our own unpublished data), while the distance between the two study sites is more than 7 km. Mist nets were used to capture adult tree sparrows in both habitats. Captured birds were placed in clean bird bags for 10–30 min to collect fecal samples. After fecal collection, the birds were weighed, ringed, and released. Sex was identified by brood patch and plumage patterns [19]. Fecal samples were preferred for this study due to their non-invasive nature and accurate representation of the gut microbiome [28]. Sterile swabs were used to collect samples, which were immediately stored in sterile tubes and frozen in liquid nitrogen for transportation. Samples were stored at −80 °C until further processing. To control for the effects of sex, body mass, and adult/juvenile, fecal samples were only collected from adult males with similar body masses (<1 g) to minimize potential effects on gut microbiome composition. Ultimately, ten fecal samples were chosen from each area (rural group: A1–A10; urban group: B1–B10) to analyze their gut microbiome.

### 2.2. Observations of Foraging Microhabitat and Foraging Events 

Observations were conducted using binoculars along randomly selected transects spanning both urban and rural habitats to document the foraging behaviors of tree sparrows. Within each habitat, two distinct foraging microhabitats were identified: rural-related and urban-related microhabitats. Sparrows observed foraging in grasslands, open woodlots, trees, farms, or similar nature-associated environments were classified as utilizing the rural-related microhabitat. Conversely, sparrows seen feeding in dust heaps, dustbins, on curbsides, or similar human-associated environments were categorized under the urban-related microhabitat. The frequency of foraging events was recorded in both rural and urban areas. The observed frequencies of foraging events in each microhabitat were compared using the Chi-square test to determine whether significant differences exist in feeding preferences between urban and rural environments.

### 2.3. DNA Extraction and 16S rRNA Gene Sequencing

DNA extraction was performed using the QIAamp Fast DNA Stool Mini Kit (Qiagen, Hilden, Germany) following the manufacturer’s instructions. The quality and quantity of the extracted DNA were assessed using a NanoDrop spectrophotometer (Thermo Fisher Scientific, Waltham, MA, USA), and the samples were stored at −20 °C until further analysis. The bacterial composition of the samples was determined by sequencing the V3–V4 region of the 16S rRNA gene. PCR amplifications were performed using the universal primers 338F (5′-ACTCCTACGGGAGGCAGCA-3′) and 806R (5′-GGACTACHVGGGTWTCTAAT-3′), containing the A and B sequencing adaptors. Sample-specific 7 bp barcodes were integrated into the primers for multiple sequencing. The PCR products were purified using the Agencourt AMPure XP system (Beckman Coulter, Brea, CA, USA) and quantified using the Qubit dsDNA HS Assay Kit (Life Technologies, Carlsbad, CA, USA). The purified amplicons were then pooled in equimolar concentrations and paired-end sequenced on an Illumina MiSeq platform according to the manufacturer’s instructions [29].

### 2.4. Statistical Analysis

The raw sequencing data, in Fastq format, is archived in GenBank (details in Data Availability Statements Section). The reads were subjected to quality filtering, trimming, and denoising. After denoising, libraries were merged, and singletons were removed. Sequence length distribution statistics were calculated using R language scripts. We used Greengenes database for bacteria taxonomy [30]. Species taxonomy annotation was performed using QIIME2 (https://github.com/QIIME2/q2-feature-classifier, accessed on 3 May 2022). The classify-sklearn algorithm [31] was employed for ASV (Amplicon Sequence Variants) feature sequence classification, and taxonomy was assigned using tax2tree 2.0 software with the default parameters for species annotation [30].

The final sequencing depth was standardized to 95% of the smallest sample sequence count (10,000 sequences per sample). Alpha diversity (within samples) and beta diversity (between samples) were calculated to evaluate the gut microbiome diversity and composition across habitats. For alpha diversity analyses, rarified ASVs were used for metrics such as Chao1, observed species, and Shannon and Simpson indices [32]. Beta diversity was analyzed using principal coordinate analysis (PCoA) to compute and visualize the weighted and unweighted UniFrac distance matrices [33]. Difference between groups was assessed with the Permutational Multivariate Analysis of Variance (PERMANOVA) test from distance matrices. The LDA effect size (LEfSe) analysis [34] was conducted to quantitatively identify biomarkers within groups. LEfSe analysis (an LDA threshold of >2) employed the non-parametric factorial Mann–Whitney U test to identify taxa that differ between groups and to test for uniformity among groups. Based on high-quality sequences, PICRUSt (Phylogenetic Investigation of Communities by Reconstruction of Unobserved States) analysis [35] was utilized to predict the functional content of 16S rRNA marker gene sequences. The KEGG database (https://www.kegg.jp, accessed on 5 May 2022) was then used to annotate the results. Functional units were obtained using the KEGG metabolic pathway database and a specific calculation method, and the abundance values of metabolic pathways were determined. The metagenomeSeq package [36] was used to identify metabolic pathways with significant differences between groups. *T* test (data of normal distribution) or Mann–Whitney U test (data of other distributions) was used to compare the differences in means between two distinct groups. In this study, we defined that group A with samples A1–A10 stands for rural groups, while group B with samples B1–B10 represents the urban group in figures of this study. Core microbiota in this study were defined as the microbiota shared by both groups inhabiting both urban and rural habitats. Statistical analyses and graphics were conducted using QIIME2 or R software (version 4.3), with *p*-values < 0.05 deemed statistically significant.

## 3. Results

### 3.1. Observation of Foraging Events 

**Figure 2:**
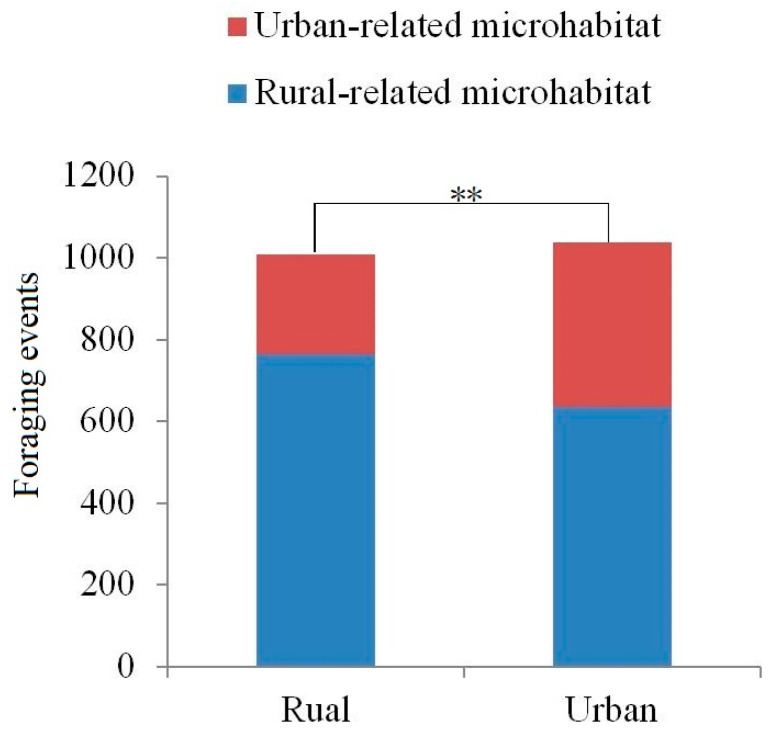
Frequencies of foraging events across two microhabitats in urban and rural settings (** indicates significant differences between groups).

The foraging events observed in each microhabitat of the two groups differed significantly, with urban tree sparrows foraging more in urban-related microhabitats (38.8% vs. 24.4%, *n* = 2047 total feeding events, χ^2^ = 49.29, *p* < 0.001; Figure 2).

### 3.2. Gut Microbiota Classification and Abundance Analysis

**Figure 3:**
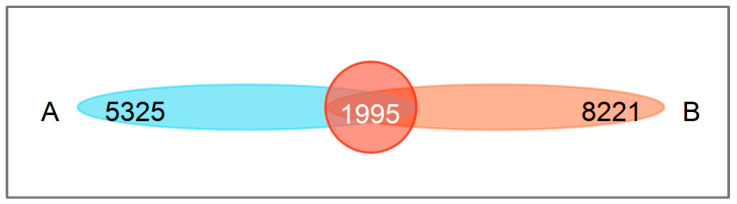
Venn diagrams showing number of shared and unique ASVs in rural group (A) and urban group (B).

Sequencing was successfully performed on 1,722,823 16S rRNA gene sequences from a total of 20 fecal samples, each containing 69,169–103,761 ASVs. The sequences varied in length from 318 to 440 bp, with an average length of 422 bp. Phylogenetic classification assigned these ASVs to 36 phyla, 120 classes, 245 orders, 453 families, and 1002 genera. It was found that the average number of ASVs and bacterial taxonomic units was slightly higher in urban sparrows compared to rural sparrows (Table 1). The Venn diagram revealed 1995 shared ASVs, representing 27.25% and 19.53% of the total ASVs in the rural and urban groups, respectively (Figure 3). Notably, the number of ASVs specific to the urban group was much higher than that of their rural counterparts (χ^2^ = 144.38; *p* < 0.001).

**Figure 4:**
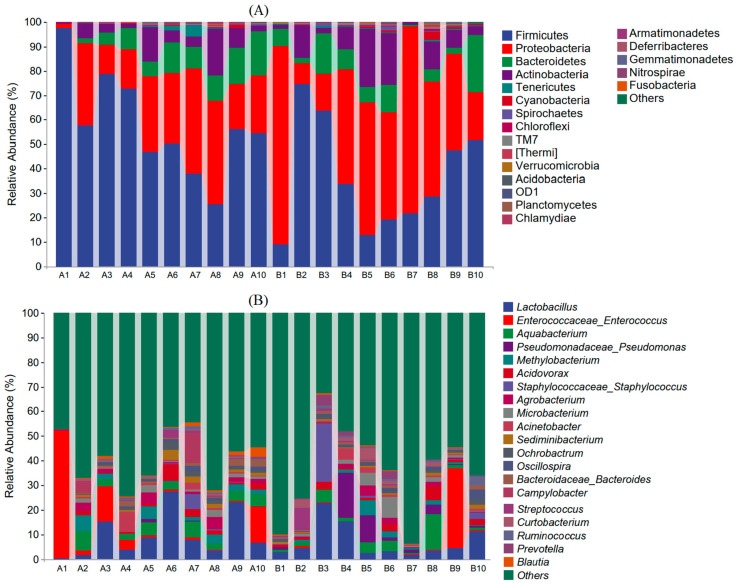
Characterization of the gut microbial community composition of each sample (A1–A10 represent rural samples and B1–B10 represent urban samples) at the phylum (**A**) and genus levels (**B**).

Figure 4A depicts the relative abundance of gut microbes in two distinct groups at the phylum level (a detailed comparison between the rural and urban groups can be found in Table S1). The rural group, composed of 33 annotated phyla, consistently exhibited four dominant phyla across all samples: Firmicutes (57.84%), Proteobacteria (25.05%), Bacteroidetes (8.66%), and Actinobacteria (6.28%). These phyla, which were represented in most samples, collectively accounted for an average of 97.83% of the microbial community. In contrast, the urban group, consisting of 35 annotated phyla, consistently displayed four similar dominant phyla across all samples: Proteobacteria (43.24%), Firmicutes (36.27%), Actinobacteria (9.46%), and Bacteroidetes (8.26%). The four phyla collectively accounted for an average of 97.24% of the microbial community. The four most dominant phyla were almost the same in the two groups (Proteobacteria, Firmicutes, Actinobacteria, and Bacteroidetes), but their relative abundance differed significantly only in two phyla (Proteobacteria and Firmicutes) between two different groups (Mann–Whitney U test: *Z* > 1.891, *p* < 0.05), with Firmicutes being the most dominant phyla in the rural group and Proteobacteria being the most dominant phyla in the urban group (Figure 4A). TM7, though ranked ninth and accounting for <0.3% in both groups, differed significantly between the rural and urban groups (rural vs. urban = 0.11% vs. 0.29%; Mann–Whitney U test: *Z* = 2.495, *p* = 0.013).

At the genus level (Figure 4B; Table S2), the gut microbiota of the rural group was mainly dominated by *Lactobacillus*, *Enterococcaceae_Enterococcus*, *Aquabacterium*, *Methylobacterium*, *Acidovorax*, *Agrobacterium*, and *Acinetobacter*. These genera were less abundant in the urban group. Conversely, the urban group exhibited higher abundances of *Pseudomonadaceae_Pseudomonas, Staphylococcaceae_Staphylococcus*, and *Microbacterium* compared to group A. These findings suggest that habitats significantly influence the composition of sparrow gut flora. At the genus level, we also observed several common potentially pathogenic bacteria, including *Staphylococcus*, *Helicobacter*, and *Shigella,* though accounting for a relatively low proportion of bacteria (<0.027%). *Staphylococcus* was found to be more abundant in the urban group (urban vs. rural: 0.027% vs. 0.007%; Mann–Whitney U test: *Z* > 1.965, *p* < 0.05).

### 3.3. Alpha and Beta Diversity Analyses

**Figure 5:**
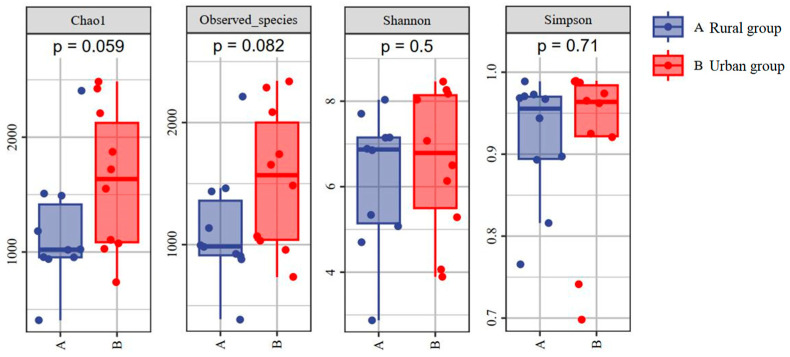
The diversity (Shannon and Simpson index) and richness (Chao1 index and observed species) of the gut microbial communities of the *Passer montanus* (groups were compared using the Mann–Whitney U test).

To further explore the impact of habitat differences, we compared the gut microbiota diversity of tree sparrows inhabiting two regions with distinct habitats. The alpha diversity of the gut microbiota was slightly higher in the urban group, although they were not statistically significant (Figure 5).

**Figure 6:**
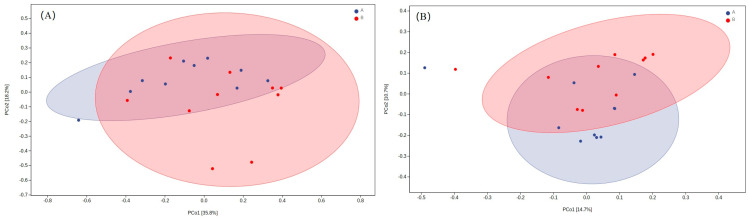
PCoA plot of samples using weighted (**A**) and unweighted (**B**) UniFrac distances between rural and urban groups.

The Chao1 index and observed species were found to be larger in the urban group compared with the rural group, and the statistical difference was close to significant. A PCoA analysis was performed to detect the beta diversity of microbial communities of the two groups. Overall, the weighted (accounting for 35.8% and 18.2% of the total variance) and unweighted (accounting for 14.7% and 10.7% of the total variance) UniFrac distances revealed the existence of a bacterial structural difference. The microbial composition between groups overlapped across nearly half of the total host species (Figure 6).

The results of the PERMANOVA based on the unweighted (*F* = 1.595; *p* = 0.005) and weighted (*F* = 2.264; *p* = 0.051) UniFrac distances confirmed that differences existed. Our findings indicate that habitat differences (urban and rural) affect the gut microbiota in sparrows.

### 3.4. LEfSe and PICRUSt2 Analysis

**Figure 7:**
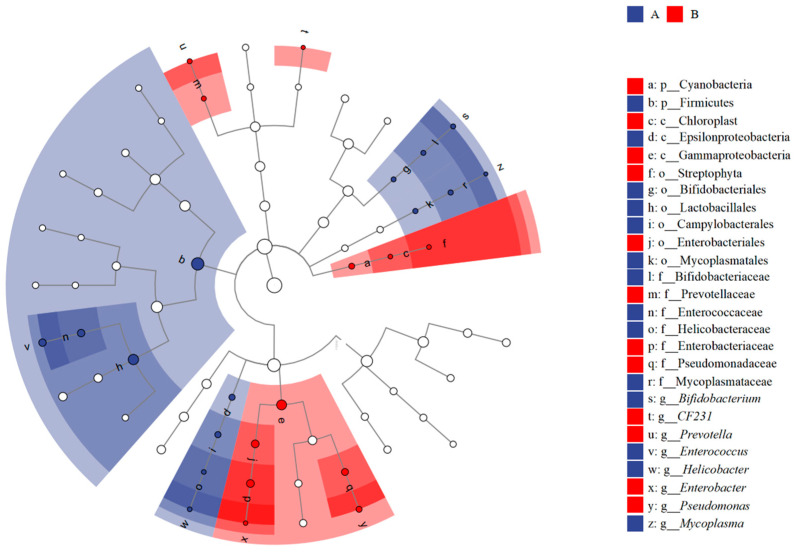
The LDA effect size of the gut microbial communities of the *Passer montanus*. The histogram shows the bacteria that were significantly greater and lower in the fecal samples of urban tree sparrows (*n* = 10) than rural tree sparrows (*n* = 10) in red and blue bars (LDA > 2).

The LEfSe analysis revealed significant differences in the relative abundance of gut bacteria between urban and rural tree sparrows. Specifically, differences were found in two phyla, three classes, six orders, seven families, and eight genera (Figure 7, LDA > 2; Figure S1).

**Figure 8:**
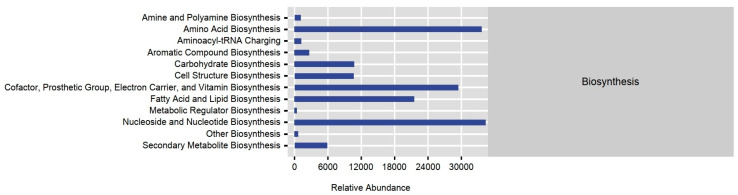
The prediction of function using a PICRUSt analysis. The horizontal coordinates stand for the abundance of functional pathways (per million KOs), the vertical coordinates stand for the functional pathways at the second-level KEGG classification, and the rightmost column stands for the first-level pathways to which these pathways belong. The average abundance of all the samples is presented.

To further investigate the function of the intestinal flora, we utilized the abundance of marker gene sequences in the samples to predict their function using the PICRUSt2 package. Our findings indicate that the biosynthesis had the highest relative abundance of genes in both groups, while other functions such as cellular processes, genetic information processing, and environmental information processing were less prominent (Figure 8).

In addition, our analysis detected statistically significant differences in three level 1 KEGG functions: biosynthesis, degradation/utilization/assimilation, and generation of precursor metabolite and energy (Table 2).

At level II KEGG ortholog functions, we observed significant differences in 13 orthologies, wherein the urban group exhibited significantly higher abundance in 12 orthologies, including amine and polyamine biosynthesis and alcohol degradation, compared to the rural group (Table 2). Furthermore, the analysis of metabolic pathways revealed significant differences in one predicted pathway number, ko03320 (PPAR signaling pathway), which was significantly increased in the urban group (*p* < 0.001).

## 4. Discussion

The intricate relationship between a host and its gut microbiota is of significant interest due to the microbiota’s role in host development, ecology, and evolution. This study harnessed high-throughput gene-based amplicon sequencing and effectively sequenced 16S rRNA gene sequences from fecal samples. The results indicate an elevated average number of ASVs and bacterial taxonomic units in urban sparrows compared to their rural counterparts, suggesting a more diverse gut microbiota in urban sparrows. This diversity may be attributed to varied diets and environmental exposures in urban locales. This study characterizes the gut microbial communities of tree sparrows in diverse habitats, providing evidence for quantitative differences in the gut microbiota between urban and rural populations. This study offers intriguing insights into the disparities in gut microbiota between rural and urban tree sparrows and the potential implications these differences may have on their physiological functions, survival strategy, and comprehensive health status.

The results reveal that Firmicutes and Proteobacteria were the top two predominant intestinal flora in both rural and urban tree sparrows, accounting for 80% of the gut microbiome (Figure 4A; Table S1). These findings align with past studies showing that Firmicutes and Proteobacteria were the top predominant intestinal flora in avian gut microbiome, such as parrots [37], kakapos [38], common diving petrels [39], and bar-headed geese [40]. Core microbes in bird guts consist of bacteria from Firmicutes, Proteobacteria, Bacteroides, and Actinomycetes [11]. Interestingly, while the four most dominant phyla (Proteobacteria, Firmicutes, Actinobacteria, and Bacteroidetes) were almost identical in both groups, their relative abundance significantly differed. Firmicutes were the most dominant in rural sparrows, whereas Proteobacteria were the most dominant in urban sparrows. The shift in dominant phyla could be attributed to different environmental factors and diet, as Firmicutes are known to play a crucial role in breaking down complex carbohydrates prevalent in the rural sparrow’s diet, while Proteobacteria are common in environments rich in simple sugars and fats, typical of urban settings [41,42]. At the genus level, the composition of gut microbiota significantly varied between the two groups, again potentially reflecting differences in diet and environmental exposure between rural and urban habitats.

The presence of potentially pathogenic bacteria in tree sparrows, such as *Staphylococcus*, *Helicobacter*, *Shigella*, and TM7, albeit in relatively low proportions, raises concerns about pathogen transmission and vector implications [9,43]. *Staphylococcus* species, which are more prevalent in urban sparrows, are implicated in a range of infections from skin conditions to sepsis [44]. Additionally, *Helicobacter* species are linked to severe gastric disorders, including gastritis and gastric cancer, with certain strains demonstrating zoonotic potential and clinical significance [45]. Furthermore, *Shigella*, found in urban sparrow intestines, is a critical foodborne pathogen known for its virulence factors such as toxin production and antibiotic resistance, contributing to diseases through contaminated mediums [46]. TM7, more abundant in urban sparrows, are obligate parasites associated with dysbiosis and inflammatory diseases but may also modulate host-induced inflammation, suggesting a complex interaction with the host’s immune response [47]. Urban sparrows exhibit a higher proportion of pathogenic bacteria, potentially enhancing their immune response and resilience against infections [48]. This adaptation could be crucial for urban sparrows in managing pathogen loads. Moreover, as vectors, birds facilitate the spread of pathogens across regions, influencing ecosystem health and nutrient cycling. This underscores the importance of monitoring urban wildlife microbiota in understanding pathogen dynamics and ecosystem impacts, particularly in light of the higher pathogen abundance in urban settings.

To a certain extent, alpha and beta diversity analyses revealed differences in the gut microbiota of tree sparrows between rural and urban groups. The alpha diversity of the gut microbiota was slightly higher in the urban group, suggesting a more diverse community of gut microbes in urban sparrows. This variation could be due to differences in diet; rural sparrows primarily consume natural foods, such as grains and insects, whereas urban sparrows have access to a broader range of food sources, including human food residues, in addition to natural foods (Figure 2). The diversity of gut microbes is often linked to the variety of food consumed by the host [49]. Venn diagrams showed similar patterns, with unique ASVs (or gut microbiota) being much higher in urban groups (Figure 3). Our findings imply that urban tree sparrows may rely on a more abundant (or unique) gut microbiota to adapt to the more varied and complex food resources and pathogen-rich environment compared to rural tree sparrows. Furthermore, studies have demonstrated that exposure to noise [50] and artificial light [51] can increase gut microbial diversity in birds, which may account for the higher bacterial richness observed in urban tree sparrows compared to rural ones. However, the observed differences in alpha diversity, although notable, were not statistically significant. This lack of significance may be attributed to the small sample size and the relatively short distance (less than 20 km) between study sites, suggesting the need for further research. The beta diversity analysis showed significant structural differences in the bacterial communities of the two groups, highlighting the influence of habitat on gut microbiota composition. Nonetheless, the presence of considerable overlap in microbial composition between the groups indicates the existence of a shared core microbiota (Figure 3), which is likely vital for fundamental physiological functions. The core microbiome is posited to consist of microbes that are consistently present over time, representing potentially indispensable genera in each individual [52].

The functional prediction of the gut flora highlighted substantial differences in the peroxisome proliferation-activated receptor (PPAR) signaling pathway between the two populations, with the urban group having a significantly higher PPAR signaling pathway. PPAR, a group of nuclear receptor proteins, regulates the expression of multiple genes involved in lipid synthesis and metabolism, immune tolerance, invasion, glucose metabolism, angiogenesis, and inflammation [53]. It is a primary regulator of differentiation, development, and cellular biological functions [54] and may serve as a metabolic stress factor, responding to varying physiological conditions by increasing the expression of enzymes involved in diverse fatty acid utilization pathways. This enhances lipid metabolism efficiency and helps maintain lipid and glucose homeostasis in the body [55].

Through LEfSe and PICRUSt2 analyses, significant differences were observed in the relative abundance of gut bacteria at various taxonomic levels and in multiple metabolic functions between the two populations. Of the 13 orthologies that showed significant differences between groups, the urban group demonstrated significantly higher abundance in 12 orthologies, such as amine and polyamine biosynthesis and alcohol degradation, compared to the rural group (Table 2). These findings, combined with the increased PPAR signaling pathway in the urban group, suggest that urban sparrows may possess enhanced abilities in synthesis, degradation, and metabolism, potentially due to their exposure to a diet high in fats and simple sugars, as well as chemical toxins. The gut flora disparity may be connected to the differences in the lipid content of the food consumed by tree sparrows in urban and rural areas. Urban sparrows are likely to ingest more human food residues, typically rich in fatty acids, whereas rural sparrows gravitate towards natural food sources with lower fatty acid contents. This dietary divergence from different microhabitats (Figure 2) may be a contributing factor to the variations in the intestinal microbes of tree sparrows between rural and urban environments. Our results corroborate that urban sparrows predominantly feed in urban-related microhabitats with human food residues (Figure 2).

## 5. Conclusions

This study has demonstrated that habitat differences, specifically between urban and rural environments, significantly impact the composition and function of gut microbiota in tree sparrows. These disparities in gut microbiota may influence the physiological functions of sparrows, potentially affecting nutrient absorption, immune function, and disease resistance. However, the specific implications of these microbial community composition differences and the effect of the PPAR signaling pathway on sparrow physiological indicators remain unclear. Further research is required to fully understand how these endogenous changes ultimately affect the adaptability and survival strategies of sparrows. Measures such as habitat management (promoting more diverse and natural habitats) and environmental monitoring (reducing harmful compounds) might improve the health of this species through gut microbiota. Additionally, our findings could potentially be leveraged in future conservation strategies, such as manipulating gut microbiota to improve the health and survival of endangered bird species across different habitats.

## Figures and Tables

**Table 1:** The average number of ASVs and different bacterial taxonomic units from tree sparrow samples in each environment.

Taxonomic Level	Rural (*n* = 10 Samples)	Urban (*n* = 10 Samples)	*Z*	*p*
ASVs	83,274.5 ± 15,105.5	89,007.8 ± 11,691.5	0.496	0.529
Phylum	20.5 ± 4.09	21.8 ± 4.66	0.565	0.579
Class	44.3 ± 11.36	51.4 ± 14.46	0.363	0.393
Order	71 ± 18.93	74.7 ± 19.56	0.762	0.796
Family	118 ± 31.87	128.4 ± 26.83	0.307	0.315
Genus	153.7 ± 54.42	177.9 ± 49.33	0.257	0.280

**Table 2:** The significant difference between urban and rural groups in KEGG orthology functions (Mann–Whitney U test).

Level 1	Level 2	Significantly Higher Abundance Group	*Z*	*p*
Biosynthesis	Amine and Polyamine Biosynthesis	Urban group	−2.343	0.019
Biosynthesis	Aromatic Compound Biosynthesis	Rural group	−1.965	0.049
Biosynthesis	Metabolic Regulator Biosynthesis	Urban group	−2.192	0.028
Biosynthesis	Other Biosynthesis	Urban group	−2.117	0.034
Degradation/Utilization/Assimilation	Alcohol Degradation	Urban group	−2.343	0.019
Degradation/Utilization/Assimilation	Aldehyde Degradation	Urban group	−2.343	0.019
Degradation/Utilization/Assimilation	Amine and Polyamine Degradation	Urban group	−2.117	0.034
Degradation/Utilization/Assimilation	Amino Acid Degradation	Urban group	−2.646	0.008
Degradation/Utilization/Assimilation	Secondary Metabolite Degradation	Urban group	−2.57	0.01
Generation of Precursor Metabolite and Energy	Entner–Duodoroff Pathways	Urban group	−2.514	0.012
Generation of Precursor Metabolite and Energy	Superpathway of Glycolysis and Entner–Doudoroff	Urban group	−2.268	0.023
Generation of Precursor Metabolite and Energy	Superpathway of Glycolysis, Pyruvate Dehydrogenase, TCA, and Glyoxylate Bypass	Urban group	−2.192	0.028
Generation of Precursor Metabolite and Energy	TCA Cycle	Urban group	−2.192	0.028

## Data Availability

The raw sequence data from this study are available upon request from the corresponding author.

## Supplementary Materials

The following supporting information can be downloaded at https://www.mdpi.com/article/10.3390/ani14233497/s1, Figure S1: The bar chart displays bacteria (from phylum to genus) represented by red and blue bars, indicating significantly higher and lower levels, respectively, in the fecal samples of rural tree sparrows (group A) compared to urban tree sparrows (group B). Table S1: A comparison of the composition and abundance of gut microbial communities in the top 20 genera between rural and urban groups. Table S2: A comparison of the composition and abundance of gut microbial communities in the top 20 phylum between rural and urban groups.
