# Supplementary material for: Comparative Study of Gut Microbiome in Urban and Rural Eurasian Tree Sparrows
Source: Animals (Basel). 2024 Dec 4;14(23):3497. doi: 10.3390/ani14233497 (PMC11640113; doi:10.3390/ani14233497)
Supplement: Supplementary file 1 [file animals-14-03497-s001.zip › animals-3301542-supplementary.pdf]

Table S1. Comparison of the composition and abundance of gut microbial communities in the top 20 phylum between rural and urban groups.

| Top 20 phylum | Rural group | Urban group | *Z* | *P* |
|---|---|---|---|---|
| Firmicutes | 57.843% | 48.968% | -1.965 | 0.049 |
| Proteobacteria | 25.051% | 33.047% | -1.890 | 0.059 |
| Bacteroidetes | 8.661% | 9.317% | -0.378 | 0.705 |
| Actinobacteria | 6.288% | 6.407% | -0.832 | 0.406 |
| Tenericutes | 0.776% | 0.815% | -1.134 | 0.257 |
| Cyanobacteria | 0.288% | 0.306% | -1.739 | 0.082 |
| Spirochaetes | 0.125% | 0.128% | -0.113 | 0.910 |
| Chloroflexi | 0.154% | 0.152% | -1.512 | 0.131 |
| TM7 | 0.113% | 0.134% | -2.495 | 0.013 |
| [Thermi] | 0.131% | 0.150% | -0.605 | 0.545 |
| Verrucomicrobia | 0.128% | 0.134% | -0.529 | 0.597 |
| Acidobacteria | 0.067% | 0.070% | -0.529 | 0.597 |
| OD1 | 0.086% | 0.092% | -0.945 | 0.345 |
| Planctomycetes | 0.043% | 0.045% | -1.209 | 0.226 |
| Chlamydiae | 0.054% | 0.054% | -0.379 | 0.704 |
| Armatimonadetes | 0.043% | 0.044% | -0.457 | 0.648 |
| Deferribacteres | 0.022% | 0.023% | -0.077 | 0.939 |
| Gemmatimonadetes | 0.011% | 0.011% | -1.268 | 0.205 |
| Nitrospirae | 0.011% | 0.012% | -0.228 | 0.820 |
| Fusobacteria | 0.015% | 0.017% | -0.383 | 0.702 |

Table S2. Comparison of the composition and abundance of gut microbial communities in the top 20 genera between rural and urban groups.

| Top 20 genera | Rural group | Urban group | *Z* | *P* |
|---|---|---|---|---|
| *Lactobacillus* | 9.898% | 10.153% | -0.756 | 0.450 |
| *Enterococcaceae_Enterococcus* | 8.965% | 3.774% | -2.343 | 0.019 |
| *Aquabacterium* | 3.987% | 4.084% | -1.285 | 0.199 |
| *Pseudomonadaceae_Pseudomonas* | 0.179% | 0.266% | -2.343 | 0.019 |
| *Methylobacterium* | 2.357% | 2.374% | -0.907 | 0.364 |
| *Acidovorax* | 1.942% | 1.994% | -0.076 | 0.940 |
| *Staphylococcaceae_Staphylococcus* | 0.702% | 0.706% | -0.870 | 0.384 |
| *Agrobacterium* | 2.032% | 2.057% | -0.832 | 0.406 |
| *Microbacterium* | 1.119% | 1.142% | -0.454 | 0.650 |
| *Acinetobacter* | 1.560% | 1.576% | -1.285 | 0.199 |
| *Sediminibacterium* | 1.291% | 1.358% | -0.907 | 0.364 |
| *Ochrobactrum* | 1.191% | 1.250% | -1.058 | 0.290 |
| *Oscillospira* | 0.763% | 0.805% | -0.151 | 0.880 |
| *Bacteroidaceae_Bacteroides* | 1.069% | 1.100% | -0.529 | 0.597 |
| *Campylobacter* | 1.717% | 1.717% | -0.937 | 0.349 |
| *Streptococcus* | 0.585% | 0.590% | -0.756 | 0.450 |
| *Curtobacterium* | 0.263% | 0.278% | -1.136 | 0.256 |
| *Ruminococcus* | 0.557% | 0.573% | -0.151 | 0.880 |
| *Prevotella* | 0.186% | 0.249% | -1.739 | 0.082 |
| *Blautia* | 0.944% | 0.980% | -1.436 | 0.151 |

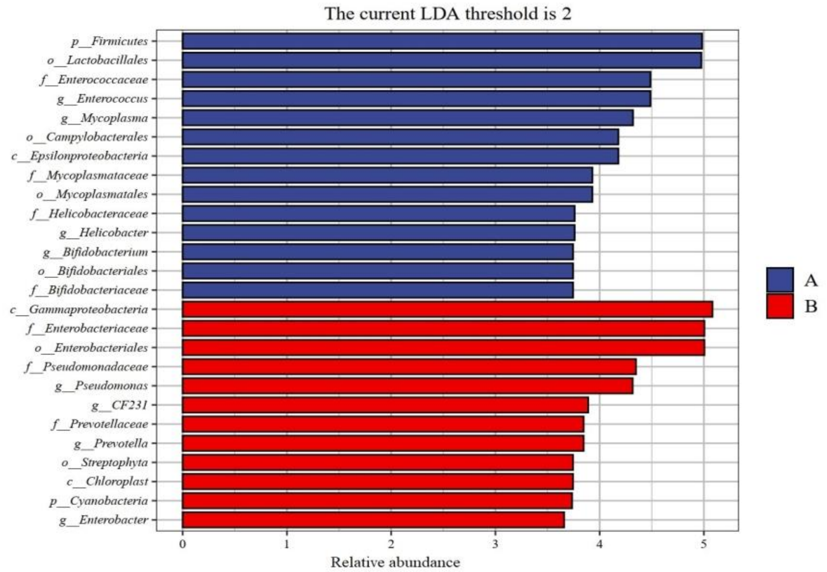


Figure S1. The bar chart displays bacteria (from phylum to genus) represented by red and blue bars, indicating significantly higher and lower levels respectively in the fecal samples of rural tree sparrows (group A) compared to urban tree sparrows (group B).
